# Cardiorespiratory fitness in working adults undergoing rehabilitation: the role of lifestyle and body composition– a cross-sectional study

**DOI:** 10.3389/fphys.2026.1806806

**Published:** 2026-05-20

**Authors:** Natalia Chróścielewska, Tomasz Chomiuk, Diana Pałasz, Katarzyna Laprus-Abramska, Artur Mamcarz, Daniel Śliż

**Affiliations:** 1 3rd Department of Internal Medicine and Cardiology, Medical University of Warsaw, Warsaw, Poland; 2 Centre of Comprehensive Rehabilitation (CKR), Konstancin Jeziorna, Poland

**Keywords:** body composition, cardiorespiratory fitness, cardiovascular health, lifestyle, pulmonary function

## Abstract

**Introduction:**

Cardiorespiratory fitness (CRF) is one of the most important predictors of mortality and morbidity. Lifestyle changes concerning diet, sleep, physical activity (PA), and substance use have a systemic impact and can prevent 80% of premature deaths and chronic diseases. CRF, being a parameter dependent on the functioning of all systems, may therefore be affected by changes in lifestyle.

**Aim:**

To examine the relationship between declared lifestyle (eating habits, alcohol use, smoking, sleep, weekly PA and body composition) and CRF.

**Methods:**

A sleep questionnaire based on Polish version of the Athens Insomnia Scale, dietary and substance survey from the PaLS study and a PA survey based on the IPAQ-SF were conducted. Body composition was assessed using a bioelectrical impedance analysis device, with particular focus on body fat percentage and skeletal muscle mass. The Åstrand-Rhyming fitness test was used to estimate VO_2max_. Spirometry was performed to assess pulmonary function, with results including FVC (% predicted), FEV_1_ (% predicted), and the FEV_1_/FVC ratio (% predicted).

**Results:**

Self-reported weekly PA was positively correlated with estimated VO_2max_ (ρ=0.23389; p= 0.0012). Night sleep duration was associated with higher estimated VO_2max_ (ρ =0.17976; p=0.0155). The frequency of consumption of plant-based protein was positively associated with estimated VO_2max_ (ρ = 0.18917; p=0.0108).). Body fat percentage was independently associated with VO_2max_ (B = -0.009, p=0.027). SMM% was independently associated with VO_2max_ (B = 0.01452, p= 0.0372).

**Conclusions:**

Self-reported weekly PA, longer sleep duration, and favorable body composition (lower body fat percentage, higher skeletal muscle mass percentage) were associated with higher VO_2max_. However, these results should be interpreted with caution due to self-reported data on lifestyle and a submaximal VO2max test.

## 1 Introduction

Cardiorespiratory fitness (CRF) is one of the most important predictors of mortality and morbidity (Blaha et al., 2016). In adults, CRF is determined by a complex interaction between genetic predisposition, health status, and lifestyle-related factors, with physical activity (PA) playing a central role. Evidence from genetic and epidemiological studies supports the concept of CRF as a clinically relevant vital sign and a robust marker of overall health (Hanscombe et al., 2021). CRF, expressed as the maximum or peak rate of oxygen consumption per kilogram of body mass (VO_2max_), reflects the integrated function of the cardiovascular, respiratory, and muscular systems and is therefore considered a key indicator of physiological health (Górski, 2019; Lang et al., 2024; LaMonte, 2022). Peak levels of CRF are typically achieved between 20 and 25 years of age, followed by a progressive decline. From approximately 35–40 years onward, this decrease is estimated at 10% per decade, although the rate of decline may vary substantially depending on lifestyle factors and body composition (Fleg et al., 2005). However, these changes can be slowed down by regular PA (Wang et al., 2014; Fleg et al., 2005).

VO_2max_ is usually assessed through gas-analyzed testing, such as Cardiopulmonary Exercise Testing (CPET), which is considered the gold standard, or non-gas-analyzed tests like Åstrand-Rhyming test or 20 Meter Shuttle Run Test, which are performed when CPET is unavailable, or there are contraindications to its performance (Raghuveer et al., 2020).

An additional important method of checking respiratory function is spirometry. Spirometry is the gold standard test for pulmonary diseases, and spirometry parameters, especially FEV_1_ and FVC, can be markers of CVD risk (Ching et al., 2019; Rydell et al., 2024; Court et al., 2023). Although studies combining spirometry results with VO_2max_ are limited, there is data suggesting that pulmonary function influences exercise capacity and a relationship between spirometry parameters and VO_2max_ (Babb et al., 1991, Babb et al., 1997; Hassel et al., 2015; Rasch-Halvorsen et al., 2019).

In recent decades, there has been a significant increase in the number of diagnosed cardiovascular, metabolic, and mental illnesses. According to the International Diabetes Federation (IDF), it is estimated that 537 million people worldwide suffer from diabetes, representing 10.5% of the total population (Magliano and Boyko, 2021). Obesity affects approximately 1 billion people, and 43% were overweight or obese (WHO, 2024). Furthermore, forecasts indicate that these numbers will continue to rise. Cardiovascular diseases are a significant group of conditions and are the leading cause of death worldwide (Di Cesare et al., 2024) and in Poland (GUS, 2021). After the age of 40, the risk of CVD increases, which is caused, among other things, by age-related arterial stiffening, metabolic changes, and cumulative lifestyle factors (Yazdanyar and Newman, 2009; Díez-Villanueva et al., 2022).

Lifestyle has a significant impact on the development of CVD diseases – poor eating habits, insufficient PA, and stress (Garg, 2025). It has been shown that lifestyle changes can prevent 80% of premature deaths and chronic diseases (Katz et al., 2018). That is why the pillars of a healthy lifestyle, according to lifestyle medicine, seem to be so important. Lifestyle medicine interventions are based on six evidence-based pillars: nutrition, regular PA, restorative sleep, stress management, avoidance of risky substances; and social connectedness.

Diet is the core pillar of lifestyle medicine, according to which the principles of proper nutrition include a whole-food, plant-predominant dietary pattern rich in vegetables, fruits, legumes, whole grains, nuts, and seeds and low in processed foods and animal products. This type of diet significantly reduces the risk of type 2 diabetes, CVDs, obesity, hypertension, and certain types of cancer (Marvasti et al., 2025). Studies have shown that a better quality diet is associated with higher CRF, particularly among young adults (Seong et al., 2020).

Sleep problems, including sleep apnea, insomnia, and circadian rhythm disturbances, as well as sleep duration and quality, influence cardiovascular health and the risk of obesity (Chasens et al., 2021). Substance use, such as tobacco, alcohol, and drugs, also negatively affects the risk of chronic diseases, and through its psychoactive effects, it worsens other aspects of lifestyle – sleep, social connections, and stress management (Naren et al., 2022). Available scientific evidence suggests that moderate alcohol consumption is associated with better physical fitness than abstinence or average higher alcohol consumption (Baumeister et al., 2018).

These pillars of lifestyle have a systemic impact. CRF, being a parameter dependent on the functioning of all systems, may therefore be affected by changes in lifestyle.

The study aims to examine the relationship between declared lifestyle, body composition, and CRF, an important predictor of morbidity and mortality. The study also examines the impact of CRF, measured using a submaximal exercise test, in combination with respiratory function assessed by spirometry, a rare approach in research that allows for a more comprehensive picture of CRF. This study is conducted on a population of professionally active people over the age of 40 undergoing rehabilitation who are at increased risk of CVD, decreased CRF, and faster, gradual deterioration of health. Although several studies to date have examined the relationship between VO_2max_ and the lifestyle of middle-aged and older adults, the number of such studies remains small, and systematic reviews indicate that there is a scarcity of data from studies combining VO_2max_ measurements with its determinants – specifically, detailed assessments of body composition and lifestyle factors such as dietary habits, sleep, substance use (Zeiher et al., 2019).

Therefore, this study may provide important public health information on beneficial lifestyle changes for CRF as a vital sign in adults.

## 2 Materials and methods

### 2.1 Participants qualification

A total of 203 individuals staying in the inpatient rehabilitation ward were enrolled in the study. Patient recruitment was carried out personally by the principal investigator. The group comprised individuals aged between 40 and 70. The average age of the respondents was 53.4, with men averaging 54.6 and women 51.8 years. 55.17% were men and 44.83% were women.

Inclusion criteria included the following: no contraindications to body composition analysis using electrical bioimpedance; current employment as a white- or blue-collar worker (with a work interruption not exceeding six months at the time of enrollment); return to work following rehabilitation; age between 40 and 70 years, and a similar functional state of the subjects (participants who undergone surgery of the lower limbs or lumbar spine due to chronic conditions during 6 months before test and who were able to perform daily cycling exercises prescribed by their physician).

Exclusion criteria included: age below 40 or above 70 years; unemployment or engagement in work other than white- or blue-collar professions; professional inactivity exceeding six months at the time of assessment; history of cancer within the previous five years; physical or mental disability; presence of chronic diseases in the exacerbation phase; period of less than six weeks after orthopedic surgery; and any medical contraindications to the performed tests.

After a preliminary eligibility assessment based on medical records by the principal investigator, participants were interviewed in person to confirm fulfillment of the inclusion criteria. Eligible individuals were subsequently invited to participate in the study. All individuals who met the inclusion criteria agreed to participate.

Participation in the study was voluntary. Written informed consent was obtained from all subjects involved in the study. The study was conducted in accordance with the Declaration of Helsinki and approved by the Bioethics Committee of the Medical University of Warsaw (KB/127/2023). The study was conducted between November 2023 and February 2025.

Of the 203 qualified individuals, 4 were unable to undergo body composition analysis. The main reason was the patient’s sudden discharge from the center. 12 people did not complete the CRF test. The main reasons were: feeling unwell during the test and abnormal blood pressure response to exercise. There were also 17 missing spirometry results due to repeated incorrect breathing maneuvers.

This work is part of a larger study that examined differences in physiological and body composition parameters between different occupational groups. The sample size was determined *a priori* using a power analysis based on the Student’s t-test for two independent means. The calculation drew upon data from the existing literature that reported statistically significant differences in BMI between blue-collar and white-collar workers (Gans et al., 2015). Although a minimum of 120 participants per group (blue-collar and white-collar workers) was required to achieve 90% power at an alpha level of 0.05, the final study sample consisted of 203 participants.

### 2.2 Questionnaire

Participants were asked about their lifestyle habits, including diet, sleep, and alcohol consumption, over the past 12 months. The questionnaires were administered by the principal investigator immediately prior to the VO_2max_ test. Participants’ eating habits were assessed using a dietary questionnaire from the PaLS study (Jodczyk et al., 2021). The questions were based on recommendations of the Polish National Institute of Public Health. The survey focused on the consumption of selected food groups, including fruits and vegetables, dairy products, plant-based protein substitutes, foods high in animal or trans fats, processed meat, sweetened beverages or fruit juices consumed instead of water, and salt. Participants indicated how often they consumed these products during a typical week. They were also asked about the frequency of alcohol consumption, caffeinated beverages, and the number of cigarettes smoked per day.

Three questions based on the Polish version of the Athens Insomnia Scale (AIS) questionnaire were asked about sleep hygiene and the occurrence of insomnia, as well as a question about the length of sleep in hours (Soldatos et al., 2000; Fornal-Pawłowska et al., 2011).

The full set of questions is available in the **Supplementary Materials**.

To assess the level of PA, a shortened version of the IPAQ-SF questionnaire was used. The data on PA are converted into MET-min/week values. Depending on the intensity of the activity, MET (metabolic equivalent) values vary – for walking (3.3 METs), moderate-intensity (4.0 METs), and vigorous-intensity activities (8.0 METs). To calculate the level of PA per week, the following calculation is performed: weekly energy expenditure (MET-min/week) = MET × duration of PA type (min) × frequency (Jodczyk et al., 2021; Balboa-Castillo et al., 2023).

Each survey was conducted in person by the principal investigator.

### 2.3 VO2max test

Cardiorespiratory fitness (CRF), expressed as estimated VO_2max_, was measured using the Åstrand–Rhyming submaximal cycle ergometer test (BTL Ergoselect 5). This test is simple, low-cost, and commonly used when direct measurement of physical performance is not feasible. Its validity for estimating VO_2_max has been confirmed in previous studies (Macsween, 2001; Vancampfort et al., 2014). Its submaximal nature allows safe and practical assessment of cardiorespiratory fitness in adults, including those who may not tolerate maximal exercise testing.

Heart rate (HR) was monitored continuously during the test and recorded every minute using a chest-mounted heart rate monitor (Polar H9). Blood pressure was measured at rest before the test using an upper-arm blood pressure monitor integrated with the ergometer and was also monitored during the test and one minute after its completion. In accordance with the recommendations of the American Heart Association (AHA), patients were asked to rest for at least 5 minutes before the measurement was taken (Whelton et al., 2018).

Absolute VO_2max_ (L/min) was estimated based on the exercise workload and the average HR. The test lasted six minutes, during which participants maintained a cycling rate of 60 ± 5 revolutions per minute. During the first two minutes, the workload was adjusted to achieve a steady HR between 120 and 175 beats per minute, corresponding to about 75% of the predicted maximum HR. The predicted maximal HR (HRmax) was calculated using the widely used equation proposed by Fox et al. (HRmax = 220 − age) (Fox and Naughton, 1972). The final workload and HR values were recorded for each participant. If the HR differed by more than five beats per minute between the fifth and sixth minute, the test duration was extended until a stable HR was reached.

Estimated VO_2max_ (mL/kg/min) was calculated by converting absolute VO_2max_ to milliliters per minute and dividing this value by body mass.

Due to the mathematical dependency between body mass and relative VO_2max_ (ml·kg^-^¹·min^-^¹), correlation analyses between BMI, BF%, SMM%, and relative VO_2max_ may be biased by shared variance. Therefore, to assess the relationship between body composition indices and aerobic capacity independently of body mass normalization, VO_2max_ was analyzed in absolute units (L·min^-^¹), adjusted only by the age correction factor according to the Astrand–Rhyming protocol.

For analyses examining the relationship between lifestyle factors (declared PA, smoking, alcohol consumption, sleep, and dietary habits) and VO_2max_, relative values (mL·kg^-^¹·min^-^¹) were used.

All VO_2max_ tests were performed by the same person to ensure methodological repeatability.

### 2.4 Pulmonary function test

Spirometry was performed according to the guidelines of the American Thoracic Society. Forced vital capacity (FVC) and forced expiratory volume in one second (FEV_1_) were measured using at least three forced expiratory maneuvers performed by each participant (Graham et al., 2019). Each participant completed three acceptable spirometry attempts. Measurements were performed in a seated erect position using a portable spirometer (EasyOne Air) and a nose clip to improve measurement accuracy. The assessed lung function parameters included FVC (% predicted), FEV_1_ (% predicted), and the FEV_1_/FVC ratio (% predicted).

### 2.5 Body composition measurement

According to many studies, body composition analysis, which provides data such as fat mass, muscle mass, and lean body mass, is a more accurate way of assessing the nutritional status of subjects and the risk of diseases. In addition, it is a more accurate supplement to the BMI values commonly used in medicine (Byker Shanks et al., 2025; Mainous et al., 2025).

Body composition was measured using the InBody 270 bioelectrical impedance analysis (BIA) device, with emphasis on body fat percentage (BF%) and skeletal muscle mass (SMM%). During the measurement, participants stood upright with their arms slightly away from their bodies and remained still and quiet until the assessment was finished.

BIA devices, including InBody devices, have demonstrated high reliability in both male and female populations, as evidenced by strong intraclass correlation coefficients (≥0.98) and low standard errors of measurement for fat mass, lean body mass, and body fat percentage (%BF) in healthy adults (McLester et al., 2020; Lee et al., 2023).

Lean mass (LM) was calculated using bioelectrical impedance analysis (BIA) as total body mass minus fat mass. Lean mass percentage (LM%) was derived by expressing LM relative to total body mass. Because findings for LM% were consistent with those observed for skeletal muscle mass percentage (SMM%), reflecting their shared physiological basis, LM% was not analyzed further.

BMI was calculated using the person’s weight in kilograms divided by the square of the person’s height in meters (kg/m^2^) (Sweatt et al., 2024).

## 3 Statistical analysis

SAS software, version 9.4 (SAS Institute Inc., Cary, NC, USA) was used for statistical analysis. Statistical significance was set at p < 0.05.

For comparisons between groups in which the distribution of the dependent variables deviated from normality, the non-parametric Kruskal–Wallis test was applied, followed by pairwise *post-hoc* comparisons with the Dwass–Steel–Critchlow–Fligner method.

To explore associations between physiological parameters and self-reported lifestyle data, the partial Spearman rank-order correlation coefficient (ρ) was used while controlling for gender. This non-parametric correlation method was chosen due to the skewed distribution of most variables.

A general linear model (GLM) was used to examine the association between body composition and physiological parameters, with sex included as a covariate. Although physiological parameters (VO_2max_ and spirometry parameters) deviated slightly from normality, the residuals of the model were approximately normally distributed, and the sample size was sufficient (n ≈ 190) to justify the use of GLM.

## 4 Results

### 4.1 Demographic data

The demographic data of the study group, including body composition and physiological parameters, are presented in **Table 1**. Continuous variables are presented as mean ± standard deviation (SD) for normally distributed data and as median with interquartile range (IQR) for non-normally distributed data.

**Table 1:** Demographic data, body composition and physiological variables stratified by gender.

	All mean ± SD	Men (n=112) mean ± SD	Women (n=91) mean ± SD
Age	53.40 ± 7.56	54.68 ± 7.62	51.84 ± 7.21
BMI	29.38 ± 4.81	29.83 ± 4.58	28.81 ± 5.05
BF%	30.87 ± 8.88	26.87 ± 7.58	35.91 ± 7.78
SMM%	38.47 ± 5.33	41.25 ± 4.48	34.96 ± 4.11
MET-min/week	2559.00 ± 2682.00	2670.00 ± 2754.00	2391.50 ± 2646.00
	All median (IQR)	Men (n=112) median (IQR)	Women (n=91) median (IQR)
Estimated VO_2max_	23.84 (8.70)	23.21 (8.37)	24.76 (7.77)
FEV1	94.00 (18.00)	93.00 (18.00)	94.00 (16.00)
FVC	98.00 (16.00)	97.00 (15.00)	98.00 (16.00)
FEV1/FVC	98.00 (10.00)	98.00 (10.00)	97.00 (9.00)

SD, standard deviation.

IQR, interquartile range.

Estimated VO_2_max in mL/kg/min.

FEV1, FVC, FEV1/FVC expressed as % predicted.

### 4.2 Association between self-reported weekly PA and CRF

Self-reported weekly PA was positively correlated with estimated VO_2max_ (ρ=0.234; p= 0.001). No significant associations were observed between spirometric parameters (FEV_1_, FVC, FEV_1_/FVC) and self-reported weekly PA. The results are shown in the **Table 2**.

**Table 2:** Partial Spearman rank correlation coefficients between self-reported weekly PA and CRF.

	Estimated VO_2max_ ρ (p-value)	FEV1 ρ (p-value)	FVC ρ (p-value)	FEV1/FVC ρ (p-value)
MET-min/week	0.2339 (**0.001)**	0.0442 (0.553)	0.0956 (0.198)	-0.0741 (0.322)

Controlled for gender.

Estimated VO_2_max in mL/kg/min.

MET-min/week refers to self-reported weekly physical activity (IPAQ-SF-derived METmin/week).

FEV1, FVC, FEV1/FVC expressed as % predicted.

ρ, partial Spearman correlation coefficient. Bold values indicate statistical significance p < 0.05.

### 4.3 Association between eating habits, smoking, caffeine consumption and CRF

In the context of the impact of caffeine consumption and smoking on estimated VO_2max_, a weak but statistically significant negative correlation was observed between smoking and FEV1 levels (ρ = -0.17, p = 0.024). In case of other parameters, no statistical significance was observed. The results are shown in the **Table 3**.

**Table 3:** Partial Spearman rank correlation coefficients between eating habits, smoking, caffeine consumption and CRF.

	Estimated VO_2max_ ρ (p-value)	FEV1 ρ (p-value)	FVC ρ (p-value)	FEV1/FVC ρ (p-value)
Smoking	-0.0565 (0.434)	-0.1660 (**0.024)**	-0.1088 (0.140)	-0.1079 (0.148)
Caffeine	0.0460 (0.539)	0.0034 (0.964)	-0.0105 (0.890)	0.0648 (0.394)
Whole grain	0.0753 (0.314)	0.0785 (0.301)	0.0615 (0.417)	0.0664 (0.383)
Dairy	0.1220 (0.102)	-0.0841 (0.267)	-0.0500 (0.510)	-0.0281 (0.712)
Fruits and vegetables	0.1216 (0.103)	-0.0052 (0.945)	0.0170 (0.822)	-0.0392 (0.607)
Meat	-0.1393 (0.061)	-0.0257 (0.735)	0.0429 (0.573)	-0.0441 (0.561)
Animal fat	0.0105 (0.889)	-0.0612 (0.421)	-0.0673 (0.377)	0.0001 (0.999)
Plant fat and fish	0.0279 (0.710)	0.1383 (0.068)	0.0723 (0.342)	0.0359 (0.638)
Plant-based protein	0.1892 (**0.011)**	0.0742 (0.328)	0.1158 (0.126)	-0.0064 (0.934)
Juices	0.1462 (**0.050)**	-0.0574 (0.451)	-0.0111 (0.884)	-0.0528 (0.489)
Salt	-0.0719 (0.336)	0.0441 (0.561)	-0.0208 (0.784)	0.0573 (0.451)

Controlled for gender.

Smoking, expressed as the self-reported number of cigarettes smoked per day.

Caffeine, self-reported number of servings consumed per week.

Products, expressed by the self-reported number of days per week of consumption.

Estimated VO_2max_ in mL/kg/min.

ρ, partial Spearman correlation coefficient.

FEV1, FVC, FEV1/FVC expressed as % predicted. Bold values indicate statistical significance p < 0.05.

The frequency of consumption of plant-based protein substitutes was positively associated with estimated VO_2max_ (ρ = 0.1892; p=0.011). Similarly, the frequency of juice consumption instead of water was positively associated with estimated VO_2_max. The results are shown in the **Table 3** together with caffeine use and smoking data.

### 4.4 Association between alcohol consumption and CRF

In the case of weekly alcohol consumption for physiological parameters deviating from the normal range, the Kruskal-Wallis test with Dwass–Steel–Critchlow–Fligner *post hoc* test was used, and the results are shown in **Table 4**. No statistically significant differences between the groups were observed either.

**Table 4:** Association between alcohol consumption and CRF.

	Estimated VO_2max_ p-value=0.147	FEV1 p-value=0.683	FVC p-value=0.706	FEV1/FVC p-value=0.691
Do not consume alcohol (n=25)	26.64 (7.72)	96.00 (19.00)	96.00 (18.00)	97.00 (9.00)
Several times a year (occasionally) (n=46)	22.75 (6.92)	94.00 (16.00)	99.00 (12.00)	97.00 (9.00)
Once a month (n=43)	23.26 (8.81)	93.00 (19.00)	98.00 (14.00)	98.50 (11.00)
Once a week (n=42)	23.91 (8.37)	94.00 (20.00)	96.50 (20.00)	97.50 (11.00)
3–4 times a week (n=21)	23.48 (9.71)	93.00 (15.00)	99.00 (17.00)	95.00 (8.00)

Median (IQR); Kruskal–Wallis test with Dwass–Steel–Critchlow–Fligner *post hoc* test.

VO_2_max in mL/kg/min.

Alcohol-expressed as the self-reported number of servings consumed.

FEV1, FVC, FEV1/FVC expressed as % predicted.

### 4.5 Association between self-reported sleep data and CRF

In the case of the relationship between sleep habits and physiological parameters, statistical significance was observed only in FEV_1_/FVC (%) sleep time categories (Kruskal–Wallis test: H = 10.94, p = 0.012). *Post hoc* analysis using the Dwass–Steel–Critchlow–Fligner test revealed a significantly higher FEV_1_/FVC (%) in the group assessing sleep time as “sufficient” (median: 98.00; IQR: 9.00) compared with the “slightly insufficient” group (median:94.00; IQR:9.00; p = 0.012), while no other pairwise differences were significant. Given the large number of comparisons and limited significant findings, only p-values are presented in **Table 5** for these associations.

**Table 5:** Association between self-reported sleep data and CRF.

	Estimated VO_2max_ p-value	FEV1 p-value	FVC p-value	FEV1/FVC p-value
Falling asleep from the moment of going to bed	0.478	0.161	0.345	0.222
Waking up during the night	0.255	0.086	0.400	0.766
Total sleep time	0.214	0.086	0.194	**0.012**
Sleep quality regardless of duration	0.399	0.213	0.145	0.358

Kruskal–Wallis test with Dwass–Steel–Critchlow–Fligner *post hoc* test was used.

VO_2max_ in mL/kg/min.

FEV1, FVC, FEV1/FVC expressed as % predicted. Bold values indicate statistical significance p < 0.05.

In examining the relationship between night sleep duration and estimated VO_2max_, night sleep duration was associated with higher estimated VO_2max_ (ρ =0.1798; p=0.016), but not with spirometry parameters, as shown in **Table 6**.

**Table 6:** Partial Spearman rank correlation coefficients between hours of sleep at night and CRF.

	Estimated VO_2max_ ρ (p-value)	FEV1 ρ (p-value)	FVC ρ (p-value)	FEV1/FVC ρ (p-value)
Estimated sleep length in hours	0.1798 (**0.016)**	0.1368 (0.070)	0.0913 (0.228)	0.1033 (0.174)

Controlled for gender.

VO_2max_ in mL/kg/min.

FEV1, FVC, FEV1/FVC expressed as % predicted.

ρ, partial Spearman correlation coefficient. Bold values indicate statistical significance p < 0.05.

### 4.6 Association between lifestyle and body composition measurements and CRF

A GLM was used to examine the association between body composition measurements and physiological parameters, with gender included as a covariate.

**Figure 1:**
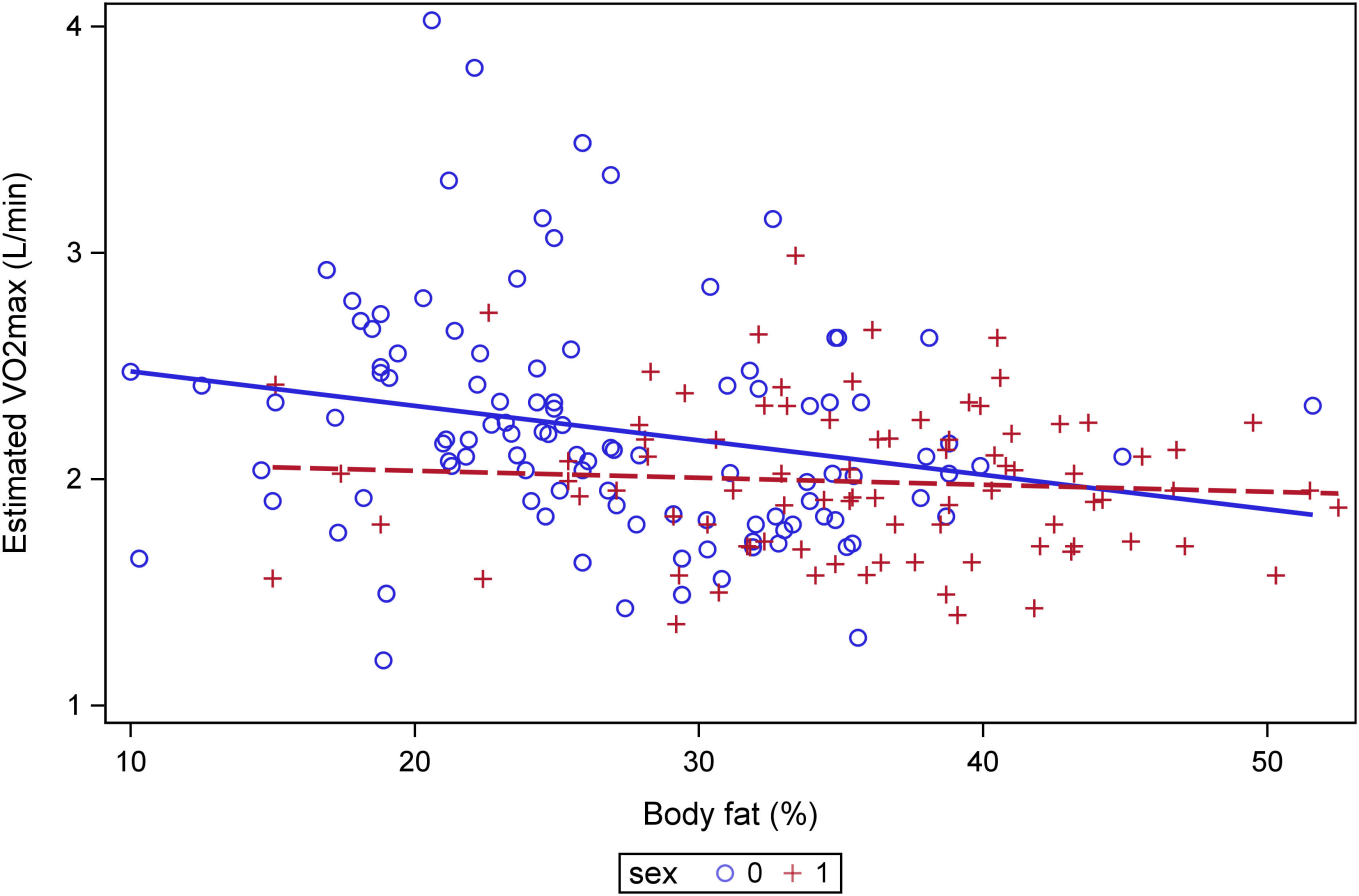
Association between body fat percentage and estimated VO_2max._ General linear model was used with gender included as a covariate.

The model was statistically significant between BF% and estimated VO_2max_ (F(2,188)=9.15, p=0.0002) and explained 8.9% of the variance in VO_2max_ (R²=0.089). Higher BF% was independently associated with lower VO_2max_ (β = -0.009, p=0.027). Sex was also a significant predictor (β = 0.14, p=0.043), with men having a higher VO_2max_ than women. A significant association between the analyzed variables, as identified by the GLM, is shown in **Figure 1**.

**Figure 2:**
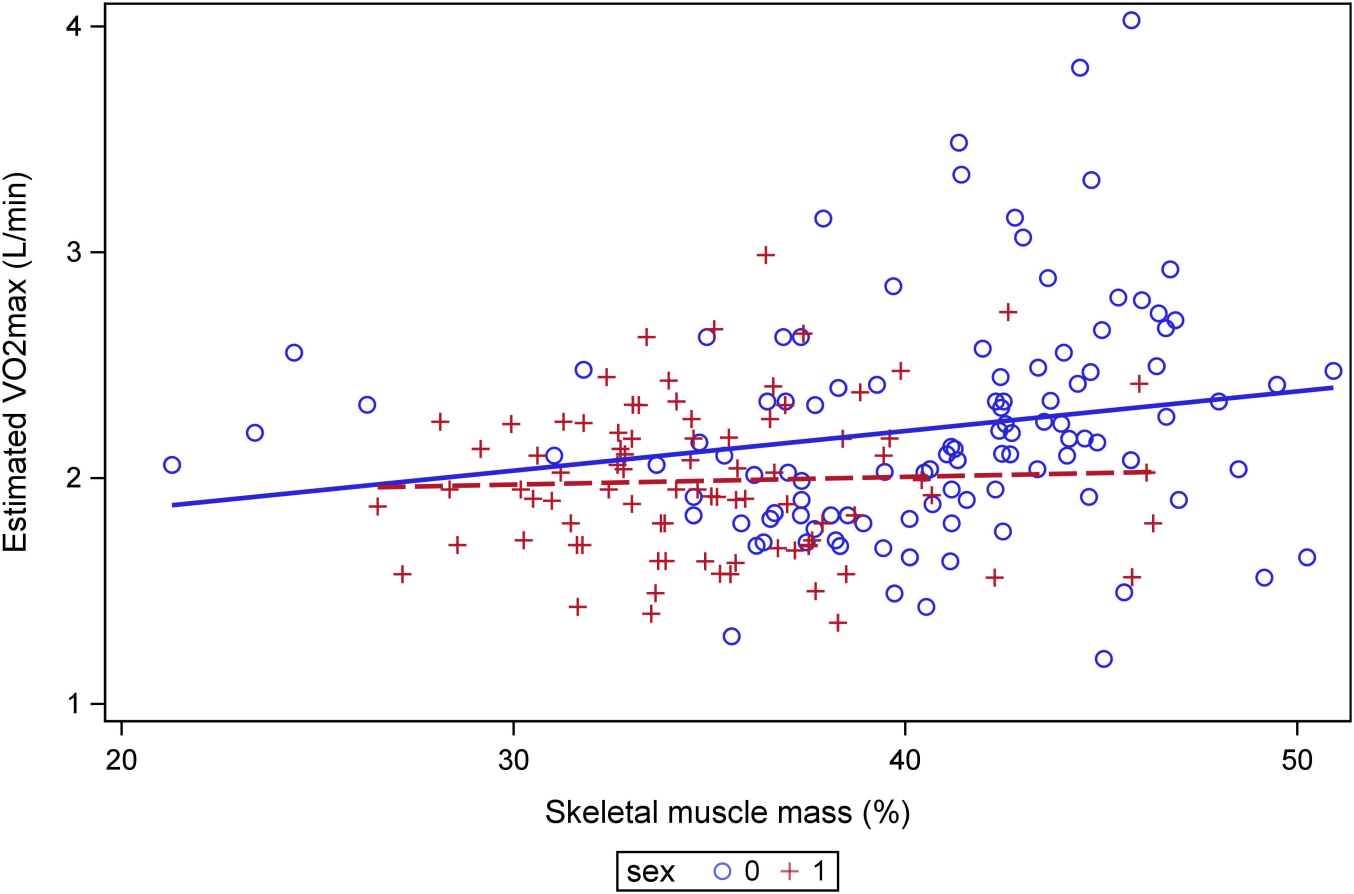
Association between skeletal muscle mass percentage and estimated VO_2max._ General linear model was used with gender included as a covariate.

In the case of examining the relationship between SMM% and VO_2max_, the model was also statistically significant for VO_2max_ (F(2,188)= 8.84, p=0.0002, R²=0.086). Higher SMM% was independently associated with higher VO_2max_ (β = 0.01452, p= 0.0372). While sex was not a significant predictor (β = 0.128, p = 0.084). A significant association between the analyzed variables, as identified by the GLM, is shown in **Figure 2**.

No significant associations were observed between SMM% and any spirometric parameters. GLM analyses showed that SMM% and sex did not significantly predict FEV_1_ (F(2,188)=0.93, p=0.337, R²=0.008; β_SMM=0.227, p=0.337; β_sex=-0.004, p=0.999), FEV_1_/FVC (F(2,188)=0.05, p=0.824, R²=0.007; β_SMM=-0.033, p=0.824; β_sex=1.521, p=0.318), or FVC (F(2,188)=2.66, p=0.105, R²=0.015; β_SMM=0.347, p=0.105; β_sex=-1.985, p=0.374).No significant associations were observed between BF% and spirometric parameters. GLM analyses showed that BF% and sex did not significantly predict FEV_1_ (F(2,188)=0.79, p=0.376, R²=0.007; β_BF=-0.118, p=0.376; β_sex=0.349, p=0.881), FEV_1_/FVC (F(2,188)=0.01, p=0.917, R²=0.007; β_BF=0.009, p=0.917; β_sex=1.400, p=0.331), or FVC (F(2,188)=1.76, p=0.186, R²=0.010; β_BF=-0.159, p=0.186; β_sex=-1.261, p=0.548) (**Table 7**).

**Table 7:** Associations between body composition variables and physiological outcomes, adjusted for sex. Only B and p-values for body composition predictors are shown.

	Estimated VO_2max_/age β (p-value)	FEV1 β (p-value)	FVC β (p-value)	FEV1/FVC β (p-value)
BMI	-0.0007 (0.915)	-0.0066 (0.975)	-0.1643 (0.400)	0.1097 (0.410)
Body fat%	**-0.0087** **(0.027)**	-0.1175 (0.376)	-0.1590 (0.186)	0.0086 (0.917)
Skeletal muscle%	**0.01450** **(0.037)**	0.2274 (0.337)	0.3475 (0.105)	-0.0325 (0.824)

General linear model with gender as covariate.

Gender as covariate.

Estimated VO_2_max in L/min.

FEV1, FVC, FEV1/FVC expressed as % predicted.

β, regression coefficient (GLM). Bold values indicate statistical significance p < 0.05.

## 5 Discussion

This study examined the associations between selected lifestyle factors and body composition with VO_2max_ and spirometric parameters. The main findings indicate that SMM%, BF%, PA, and sleep duration were associated with VO_2max_, while most other lifestyle factors showed no significant relationships. SMM% showed the strongest positive association with VO_2max_, while BF% had a weaker negative effect. In addition, higher levels of self-reported weekly PA and declared longer night-time sleep were associated with higher estimated VO_2max_. Given the self-reported data on eating habits, sleep and substance use and the associated recall bias and social desirability bias, the study results should be interpreted with caution and primarily as a basis for formulating hypotheses. Moreover, PA was assessed using the IPAQ-SF, which is known to often overestimate weekly activity levels. Similarly, VO_2max_ was estimated using the submaximal Astrand–Rhyming test, which, while practical, introduces inherent measurement error. Additionally, age may influence VO_2max_ and should be considered when interpreting the results. In this study population, the median VO_2max_ for participants aged 40–70 years was 23.84 mL·kg^-^¹·min^-^¹, with a mean age of 53.4. This value is relatively low compared to reference data for healthy middle-aged adults, suggesting reduced aerobic capacity in this cohort (Van der Steeg and Takken, 2021). While VO_2max_ is strongly correlated with overall CRF, the submaximal Astrand–Rhyming test used in this study introduces inherent measurement error and may slightly underestimate true maximal capacity and their clinical or physiological relevance may be limited. Therefore, the strength of the observed associations should be interpreted with caution, particularly for statistically significant but small effect sizes. These limitations highlight the need for further studies using objective measures of both PA and maximal CRF to confirm and refine the associations observed in this population. Nevertheless, these findings indicate that even in apparently healthy adults, age and body composition can substantially influence aerobic fitness, highlighting the importance of interventions targeting PA, sleep, and muscle mass to maintain or improve CRF in this age group.

VO_2max,_ as a primary indicator of CRF, is a parameter that comprehensively reflects the functioning of the cardiovascular, muscular and respiratory systems, including cardiac output, oxygen transport and oxygen utilization in peripheral tissues (Heinonen, 2025; Grassi et al., 2021). A higher VO_2max_ is associated with greater cardiac output, increased mitochondrial density in skeletal muscle, and improved vascular function (Boushel et al., 2011; Buscemi et al., 2013). Lifestyle factors such as PA, diet and sleep can influence these mechanisms by affecting inflammation and cardiovascular function (Chen et al., 2021). Body composition also plays a significant role, as higher body fat mass may impair oxygen utilization efficiency, whilst greater skeletal muscle mass supports aerobic metabolism (Phoemsapthawee and Phoemsapthawee, 2025). These physiological mechanisms provide a framework for interpreting the associations observed in the present study between lifestyle factors, body composition, and VO_2max_. Importantly, VO_2max_ is recognized as a strong predictor of morbidity and all-cause mortality, reflecting the overall performance of numerous physiological systems (Lang et al., 2024; Kodama et al., 2009). Therefore, even small differences in VO_2max_ values may have implications for long-term health outcomes.

### 5.1 Body composition and VO_2max_

The results of our study regarding the relationship between body composition and VO_2max_ are consistent with previous scientific reports and well-established physiological mechanisms. Goran et al. point to the greater role of fat-free mass in shaping VO_2max_ than fat mass (Goran et al., 2000). In turn, a study by Bellissimo et al. observed that individuals classified as normal-weight-obese had lower CRF compared to lean individuals, despite having a similar diet quality (Bellissimo et al., 2020).

Considering the above reports and research on muscle tissue physiology, it can be suspected that SMM% plays a significant role in shaping VO_2max_. In addition, skeletal muscles also play an important role in preventing many lifestyle diseases, such as CVD (Xu et al., 2023; Lu et al., 2023). Skeletal muscle secretes a variety of myokines, especially during exercise, such as fibroblast growth factor-21 (FGF-21), follistatin-like 1, brain-derived neurotrophic factor (BDNF), interleukin-6 (IL-6), and myonectin, that have both local and systemic health-promoting effects. These chemicals promote uptake and lipolysis of free fatty acids in skeletal muscle and the liver, increase glucose uptake, have neurocognitive benefits, improve endothelial function, promote angiogenesis, and protect against ectopic fat deposition (Chen et al., 2021; Kim and Kim, 2020). This may explain why SMM% showed the strongest association with VO_2max_ in our study.

### 5.2 Self-reported physical activity and VO_2max_

The observed positive association between PA and VO_2max_ is consistent with well-established evidence showing that regular PA has a health-promoting effect on the entire body and contributes to the improvement of many organ functions (Chomiuk et al., 2024) (Wang and Ashokan, 2021). Decreased PA is strongly associated with increased obesity (Nunn et al., 2010). An inverse correlation is observed between PA, body mass index (BMI), waist-to-hip ratio (WHR), and waist circumference. Additionally, a reduction in fat mass leads to increased adiponectin levels and improved cytokine profiles, changes that are associated with metabolic syndrome and the development of insulin resistance. In the study by Venojarvi et al, it was observed that improved insulin sensitivity was associated with improved peak oxygen uptake and physical performance (Venojärvi et al., 2022). Considering the above observations, attention should be paid to the role of strength training, as SMM% is influenced to a large extent by physical exercise with appropriate load, and not only by diet (Da Silva Gonçalves et al., 2023; Feng et al., 2024).

### 5.3 Sleep and VO_2max_

In terms of the number of hours of sleep at night in this study, it should be noted that the maximum number of hours of sleep in the study was 10, which was declared by one person. The largest number of people (n=79) declared that they sleep 7 hours, 40 people declared 8 hours of sleep, and 47 declared 6 hours. The quality and length of sleep have a systemic effect and may therefore influence changes in VO_2max_. Short sleep duration can disrupt carbohydrate metabolism, the hypothalamic-pituitary-adrenal axis, and sympathetic nervous system activity, which are linked to increased waist circumference and weight gain (Cappuccio and Miller, 2017). Elevated cortisol levels are also associated with higher blood pressure. Sleeping less than 6 hours raises the risk of metabolic syndrome (Che et al., 2021), while in adults, sleeping more than 10 hours negatively affects the lipid profile (Kim et al., 2018). Antues et al. observed that participants reporting good sleep quality presented higher values of VO_2max_ which is consistent with the results of the study (Antunes et al., 2017).

In summary, these findings suggest that PA, body composition and sleep may interact, influencing aerobic capacity via shared physiological pathways, including metabolic regulation, inflammation and hormonal balance. PA contributes to an increase in SMM, which improves oxygen utilization and insulin sensitivity. Skeletal muscle exerts an anti-inflammatory effect, benefiting the cardiovascular and nervous systems as well as blood glucose levels. Insufficient sleep, on the other hand, negatively affects carbohydrate metabolism and may lead to chronic inflammation. These factors may therefore act synergistically, shaping aerobic capacity and protecting against the onset of metabolic disorders.

### 5.4 Non-significant lifestyle factors and potential explanations

No statistically significant differences were observed in the context of stimulants (smoking and alcohol consumption). The lack of significant results may be related to the likelihood that individuals are responding in a socially desirable manner and to methodological problems associated with estimating alcohol consumption, as described in previous studies (Davis et al., 2010; Dawson, 2003). In the study, no one reported drinking alcohol daily, while the largest group (n=51) reported drinking several times a year. Despite very little data on the relationship between alcohol consumption and VO_2max_, available reports suggest a negative relationship between smoking and heavy drinking on VO_2max_ (Montoye et al., 1980). Smoking also negatively affects VO_2max_ and Spicuzza et al. indicate that giving up tobacco products improves CRF in people who previously smoked (Spicuzza et al., 2025). In a study by Suminski et al., it was observed that heavy smokers are most at risk of low VO_2max_ (Suminski et al., 2009).

In the context of self-reported eating habits, only the consumption of plant-based protein substitutes was found to have a significant association with VO_2max_. Higher consumption of such products was associated with higher VO_2max_. It should be noted that these results should be interpreted with caution, as the data were self-reported, which imposes a bias associated with the inaccuracy of this type of study. Scientific reports on the impact of diet on VO_2max_ are limited, but Ariazza et al. observed that following the principles of the Mediterranean diet is associated with higher VO_2max_ (Santiago-Arriaza et al., 2025). Legumes, which are a plant-based protein substitute, are an important part of the Mediterranean diet (Fresán et al., 2018). However, the limited number of studies and differences in methodology prevent drawing firm conclusions. In turn, the findings suggest the benefits of a low-carbohydrate, high-fat diet on CRF (Prins et al., 2023), but the evidence remains inconsistent. Therefore, our findings primarily highlight an association with plant-based protein consumption rather than a definitive dietary effect on VO_2max_.

The lack of significant associations between most dietary habits and VO_2max_ can be explained by several factors. Firstly, self-reported dietary data may be subject to recall bias, particularly when participants are required to estimate average portion sizes. Secondly, the face-to-face nature of the survey may have increased the likelihood of responses conforming to social expectations, particularly regarding alcohol consumption and overall diet quality. Furthermore, the relatively homogeneous nature of the study population may have limited the variability in dietary patterns, which further reduced the likelihood of detecting significant associations.

These findings highlight the need for future studies to employ more detailed and objective methods of dietary assessment, as well as to include more diverse populations, in order to better understand the relationship between nutrition and cardiorespiratory fitness.

### 5.5 Spirometry and lifestyle factors

In terms of spirometric parameters, only a small but statistically significant negative correlation was observed between smoking and FEV1 levels, so smoking alone explains very little of the variability in FEV1. Previous studies have shown that smoking leads to poorer spirometry results compared to non-smokers (Kumar et al., 2022). In turn, a study by Dugral et al. observed that smoking improved lung function in young smokers (Dugral and Balkanci, 2019). Similarly, Regan et al. observed that lung disease and impaired lung function were common among smokers without spirometric COPD (Regan et al., 2015). In addition, significantly higher FEV_1_/FVC was observed in the group assessing sleep time as ‘sufficient’ compared with the ‘slightly insufficient’ group. These differences may fall within the measurement error of the test and self-reported sleep data. Among lifestyle-related factors, studies point to the role of obesity in the deterioration of respiratory function (Dixon and Peters, 2018). The weak correlation between lifestyle variables and spirometric parameters may indicate that the lifestyle parameters studied do not have a significant impact on changes in pulmonary function. The population selected for the study was generally healthy, so it can be assumed that there were no differences depending on spirometric parameters.

In summary, these findings suggest that lifestyle factors may have a greater impact on aerobic capacity than on spirometric parameters in populations of relatively healthy individuals.

## 6 Strengths and limitations

This study presents several important strengths that enhance the reliability and applicability of its findings. First, it assesses CRF — a well-established predictor of morbidity and mortality — using a method that is both cost-effective and easily accessible. This enhances the potential for future implementation in various public health settings. Furthermore, there are few studies examining the relationship between body composition and CRF, mainly in populations of athletes or young people. Examining the relationship between body composition and physiological parameters, particularly the important estimated VO_2max_, provides significant information for public health and may help to identify directions for the prevention of lifestyle diseases. What is more, the study examines the impact of CRF measured using a submaximal exercise test together with respiratory function using spirometry, which is a rare approach in research and allows for a more comprehensive picture of CRF. Finally, procedures such as participant qualification, questionnaire administration, and VO_2max_ testing were performed by the same trained researcher. This approach minimized interrater variability and improved the internal validity of the collected data.

This study has some limitations. The first limitation of this study relates to the generalizability of its findings. While the results reflect the experiences of Polish citizens, they may not apply to individuals from other countries. Limitations include its cross-sectional design, which prohibits causal inferences to future disease incidence. Furthermore, data from the survey on eating habits, smoking, alcohol, caffeine consumption and sleep, which is based on respondents’ self-assessment, is also an important limitation. The low reliability of survey data suggests that conclusions from the research should be drawn with great caution, and primarily treated as a basis for formulating hypotheses. Furthermore, an important limitation of this study is that it relies on self-reported PA assessed using the IPAQ-SF questionnaire. This tool may overestimate PA levels and does not distinguish between PA performed at work and PA performed during leisure time. Finally, despite detailed inclusion criteria aimed at selecting generally healthy subjects at a similar functional level, postoperative condition, although well-functioning, ready to come back to work, may constitute a bias in the study. Therefore, the findings of this study may be of greatest relevance to middle-aged and older adults who are undergoing rehabilitation and preparing to return to work. Caution should therefore be exercised when generalizing these findings to generally healthy, working-age adults.

## 7 Conclusions

Self-reported weekly PA, longer sleep duration, and favorable body composition (lower BF%, higher SMM%) were associated with higher VO_2max_, which is an important predictor of cardiovascular disease and mortality.

Importantly, most self-reported dietary habits, as well as alcohol and caffeine consumption, were not significantly associated with VO_2max_ or spirometric parameters in this study. This suggests that, within this population, PA, sleep, and body composition may represent more prominent modifiable factors related to CRF than specific dietary behaviors or moderate substance use.

These findings should be interpreted with caution due to the self-reported nature of lifestyle data and the use of a submaximal method for VO_2max_ estimation, which may introduce measurement error. The lack of significant associations for several lifestyle variables may also reflect limitations of the cross-sectional design or the accuracy of self-reported data.

The topic requires further research, particularly on the impact of eating habits, sleep, and stimulants on VO_2max_ and pulmonary function, as there are still few studies examining these associations.

## Data Availability

The raw data supporting the conclusions of this article will be made available by the authors, without undue reservation.
