# Supplementary material for: Cardiorespiratory fitness in working adults undergoing rehabilitation: the role of lifestyle and body composition– a cross-sectional study
Source: Front Physiol. 2026 May 20;17:1806806. doi: 10.3389/fphys.2026.1806806 (PMC13229796; doi:10.3389/fphys.2026.1806806)
Supplement: Supplementary file 1 [file Table1.docx]

Supplementary matierial

**Dietary survey from the PaLS**

Please estimate how many days in an average week:

1. Do you consume up to three portions of whole grain products? (90g/day) (In manuscript listed as: Whole grain)

2. Do you consume up to 400g of vegetables and fruit? (In manuscript: Fruits and vegetables)

3. Do you consume up to 2 glasses of unsweetened milk or other dairy products? (one glass of milk corresponds to a glass of yoghurt, kefir, buttermilk or approx. 6 thick slices of white cheese) (In manuscript: dairy)

4. Do you consume processed meat products? (Processed meat includes frankfurters, sausages, ham, etc.) (In manuscript: Meat)

5. Do you replace meat with plant-based protein products (plant-based proteins, i.e. legumes: beans, chickpeas, soybeans, peas, lentils, broad beans) and nuts? (in manuscript listed as: Plant-based protein)

6. Are the sources of fat in your diet animal fats or partially hydrogenated vegetable fats found in biscuits, chocolate bars, savoury snacks and fast food, among other things? (In manuscript: animal fat)

7. The source of fat in your diet was vegetable fats (i.e. rapeseed oil, olive oil) or fish? (In manuscript: Plant fat and fish)

8. Did you reach for sweet drinks or fruit juices instead of water? (In manuscript: juices)

9. Did you add salt to your food? (In manuscript: salt)

**Stimulants survey**

1. On average, how many cigarettes did you smoke per day? * If you do not smoke cigarettes at all, mark ‘0’.
2. How often have you consumed a serving of alcoholic beverage during the past year while the pandemic has been ongoing? One serving (10 g of ethanol) is approximately 250 ml of 5% beer, 30 ml of 40% vodka, or 100 ml of 12% wine.

- Do not consume alcohol
- Several times a year (occasionally)
- Once a month
- Once a week
- 3-4 times a week
- Every day

1. On average, how many servings of caffeinated beverages do you consume per week? Portion examples:

Espresso coffee (50 ml)

Ground coffee/instant coffee (200 ml)

Cappuccino (200 ml)

Tea (200 ml)

Cappuccino (200 ml)

Tea (200 ml)

Energy drink (250 ml)

Cocoa (200 ml)

Sweet caffeinated drinks, e.g. Coca-Cola (1 can/330 ml)

**Sleep survey:**

1. Falling asleep from the moment of going to bed:

- quickly
- slightly delayed
- delayed
- very delayed or sleepless throughout the night

1. Waking up during the night
   - does not occur

- occasionally
- often
- very often or insomnia throughout the night

1. Total sleep time

- Completely insufficient or insomnia throughout the night
- Clearly insufficient
- Slightly insufficient
- Sufficient

1. Sleep quality regardless of duration

- Completely unsatisfactory
- clearly unsatisfactory
- slightly unsatisfactory
- satisfactory

1. Estimated sleep length in hours:
